# Short-acting β_2_-agonist prescription patterns for asthma management in the SABINA III primary care cohort

**DOI:** 10.1038/s41533-022-00295-7

**Published:** 2022-09-29

**Authors:** David Price, Kerry Hancock, Joseph Doan, Sri Wahyu Taher, Chakaya J. Muhwa, Hisham Farouk, Maarten J. H. I. Beekman

**Affiliations:** 1 Observational and Pragmatic Research Institute, Singapore, Singapore; 2 Centre of Academic Primary Care, Division of Applied Health Sciences, University of Aberdeen, Aberdeen, UK; 3 Chandlers Hill Surgery, Happy Valley, SA Australia; 4 HealthPlus Medical Centre, Kogarah, NSW Australia; 5 Simpang Kuala Health Clinic, Alor Setar, Kedah Malaysia; 6 Department of Medicine, Therapeutics, Dermatology and Psychiatry, Kenyatta University, Nairobi, Kenya; 7 AstraZeneca, Dubai, UAE; 8 AstraZeneca, The Hague, The Netherlands

**Keywords:** Asthma, Therapeutics

## Abstract

Short-acting β_2_-agonist (SABA) prescriptions and associated outcomes were assessed in 1440 patients with asthma from the SABA use IN Asthma (SABINA) III study treated in primary care. Data on asthma medications were collected, and multivariable regression models analysed the association of SABA prescriptions with clinical outcomes. Patients (mean age, 47.9 years) were mostly female (68.6%); 58.3% had uncontrolled/partly controlled asthma and 38.8% experienced ≥1 severe exacerbation (reported in 39% of patients with mild asthma). Overall, 44.9% of patients were prescribed ≥3 SABA canisters (over-prescription) and 21.5% purchased SABA over-the-counter. Higher SABA prescriptions (vs 1−2 canisters) were associated with significantly decreased odds of having at least partly controlled asthma (6–9 and 10–12 canisters) and an increased incidence rate of severe exacerbations (10–12 and ≥13 canisters). Findings revealed a high disease burden, even in patients with ‘mild’ asthma, emphasising the need for local primary care guidelines based on international recommendations.

## Introduction

Asthma is one of the most common chronic conditions affecting over 300 million people worldwide^1,2^. Despite the availability of a range of effective treatment options, asthma continues to impose a significant socioeconomic burden on patients and healthcare systems worldwide^3^. Although healthcare policies and evidence-based treatment recommendations at both national and international levels are available to guide clinical practice, asthma remains uncontrolled in a considerable proportion of patients^4,5^.

Among all healthcare practitioners (HCPs), primary care physicians (PCPs) represent the first point of contact for most patients with asthma^6^. Consequently, they play an essential role in the holistic management of asthma^7,8^ and are uniquely positioned to exert a major influence on asthma care^3,6^. Indeed, the World Health Organization (WHO) recognises that healthcare systems with strong primary care services are crucial to improve treatment outcomes and deliver comprehensive care^9^. However, the availability of good-quality primary care remains highly variable, with the WHO reporting that the average health spending per person in 2017 was approximately 70 times lower in low income countries (US $41) than in high income countries (US $2937)^10^. Moreover, results from a systematic review reported that in 18 countries representing approximately 50% of the global population, PCPs spent 5 min or less per consultation with their patients^11^. In addition, PCPs are often faced with a multitude of challenges, particularly diagnostic uncertainties in patients with mild asthma^12,13^ or those with normal lung function parameters^14^. Additionally, implementation of evidence-based recommendations in the primary care setting may be hindered by factors such as non-availability of diagnostic resources in many geographical regions, limited time for in-depth diagnostic assessments, and lack of specific primary care guidelines^6,7,15,16^.

Inappropriate use of asthma medications^17^, in the form of excessive use of short-acting β_2_-agonists (SABAs), possibly combined with underuse of inhaled corticosteroids (ICS)^16,18–20^, remains a major challenge in respiratory care, contributing to poor treatment outcomes^21^. Therefore, following a landmark update in 2019^22^, the Global Initiative for Asthma (GINA) no longer recommends as-needed SABA without concomitant ICS for patients ≥12 years of age^3^. These updated treatment recommendations largely impact PCPs who represent the frontline of patient management. However, PCPs are often not familiar with GINA^23^, and there is often a time lag between revised GINA recommendations and updates to local guidelines. This may contribute to clinical practices not being aligned with the latest treatment recommendations, resulting in PCPs prescribing SABAs to patients at the time of asthma diagnosis, thereby delaying the initiation of regular preventer medication^24^. In addition, most patients with asthma have mild disease and are therefore commonly prescribed SABA-only treatment^25^. However, all patients, including those with mild asthma, are at risk of exacerbations^3^, with clinical trial data reporting that patients with mild asthma using as-needed SABA experience a 60% higher rate of moderate-to-severe exacerbations than those receiving ICS-containing treatment^26^. Therefore, a detailed understanding of SABA prescription practices across asthma severities in primary care, including the potential impact of SABA over-the-counter (OTC) purchase, would be of considerable value, particularly in less well-resourced countries where current data on SABA use are lacking.

To continue to assess the global extent of SABA use and its clinical consequences, the SABA use IN Asthma (SABINA) III (International) study was initiated in 24 countries across 5 continents in 8351 patients treated by both PCPs and specialists. Results reported that more than a third of patients with asthma were over-prescribed SABAs (≥3 canisters) in the 12 months prior to study entry^27^. Moreover, SABA over-prescription was associated with poor asthma-related health outcomes^27^. To better understand how asthma is managed globally in the primary care setting, we assessed prescriptions of SABA and other asthma medications (including ICS-containing treatments and oral corticosteroids [OCS]), OTC purchases of SABA, and clinical outcomes associated with SABA prescriptions in the cohort of patients from the SABINA III study who were treated by PCPs.

## Results

### Study population and patient characteristics

Of the 8351 patients in the SABINA III study, 17.2% (*n* = 1440) were managed exclusively by PCPs. Patients had a mean (standard deviation [SD]) age of 47.9 (16.73) years (Table 1). The majority of patients were female (68.6%), had a body mass index (BMI) of ≥25 kg/m^2^ (63.8%), and had never smoked (78.7%). Over one-third of patients (35.5%) reported no healthcare reimbursement.

**Table 1:** Demographics and baseline clinical characteristics of the SABINA III PCP population by investigator-classified asthma severity.

Characteristics	Primary care (*n* = 1440)		
	Investigator-classified mild asthma^a^ (*n* = 743)	Investigator-classified moderate-to-severe asthma^a^ (*n* = 695)	All (*n* = 1440)
Age, years			
Mean (SD)	45.8 (16.80)	50.2 (16.38)	47.9 (16.73)
Median (min–max)	47.0 (12.0–95.0)	51.0 (12.0–93.0)	49.0 (12.0–95.0)
Age group, years			
12–17	53 (7.1)	17 (2.4)	70 (4.9)
≥18–54	443 (59.6)	399 (57.4)	844 (58.6)
≥55	247 (33.2)	279 (40.1)	526 (36.5)
Sex			
Female	535 (72.0)	452 (65.0)	988 (68.6)
Male	208 (28.0)	243 (35.0)	452 (31.4)
BMI, kg/m^2^			
Mean (SD)	27.7 (6.44)	28.1 (6.55)	27.9 (6.49)
Median (min–max)	26.7 (12.6–57.0)	27.3 (16.3–75.0)	26.9 (12.6–75.0)
BMI group, kg/m^2^			
<18.5	32 (4.3)	15 (2.2)	47 (3.3)
≥18.5–24.9	241 (32.4)	232 (33.4)	474 (32.9)
≥25.0–29.9	247 (33.2)	230 (33.1)	477 (33.1)
≥30.0	223 (30.0)	218 (31.4)	442 (30.7)
Education level			
Not established	28 (3.8)	60 (8.6)	88 (6.1)
Primary school	146 (19.7)	62 (8.9)	208 (14.4)
Secondary school	200 (26.9)	125 (18.0)	325 (22.6)
High school	166 (22.3)	151 (21.7)	318 (22.1)
University and/or post-graduate education	203 (27.3)	297 (42.7)	501 (34.8)
Healthcare insurance/medication funding			
Not reimbursed	320 (43.1)	191 (27.5)	511 (35.5)
Partially reimbursed	152 (20.5)	196 (28.2)	348 (24.2)
Fully reimbursed	258 (34.7)	281 (40.4)	539 (37.4)
Unknown	13 (1.7)	27 (3.9)	42 (2.9)
Smoking status history			
Active smoker	27 (3.6)	63 (9.1)	91 (6.3)
Former smoker	97 (13.1)	119 (17.1)	216 (15.0)
Never smoker	619 (83.3)	513 (73.8)	1133 (78.7)

Data are presented as *n* (%) unless otherwise specified. ^a^Investigator-classified asthma severity was guided by GINA 2017 treatment steps. Investigators were guided by GINA 2017 treatment steps, either in the study protocol or via a pop-up window in the eCRF.

*BMI* body mass index, *eCRF* electronic case report form, *GINA* Global Initiative for Asthma, *max* maximum, *min* minimum, *PCP* primary care physician, *SABINA* SABA use IN Asthma, *SD* standard deviation.

### Disease characteristics

The mean duration of asthma was 17.2 years (Table 2). Investigator-classified mild (GINA treatment steps 1–2) and moderate-to-severe (steps 3–5) asthma was reported in 51.7 and 48.3% of patients, respectively. Overall, 41.2% of patients had no comorbidities, with 41.4% reporting 1–2 comorbidities,. Patients reported a mean (SD) of 1.0 (2.42) severe asthma exacerbation in the 12 months preceding study initiation, with 38.8 and 12.3% of patients experiencing ≥1 and ≥3 severe exacerbations, respectively.

**Table 2:** Asthma characteristics of the SABINA III PCP cohort according to investigator-classified asthma severity.

Asthma characteristics	Primary care (*n* = 1440)		
	Investigator-classified mild asthma^a^ (*n* = 743)	Investigator-classified moderate-to-severe asthma^a^ (*n* = 695)	All (*n* = 1440)
Asthma duration, years			
Mean (SD)	17.9 (14.78)	16.5 (13.91)	17.2 (14.37)
Median (min–max)	13.0 (1.0–73.0)	13.0 (1.0–70.0)	13.0 (1.0–73.0)
Number of severe asthma exacerbations 12 months before the study visit			
Mean (SD)	1.1 (2.99)	0.9 (1.60)	1.0 (2.42)
Number of severe asthma exacerbations 12 months before the study visit			
0	453 (61.0)	428 (61.6)	882 (61.3)
1	129 (17.4)	130 (18.7)	259 (18.0)
2	59 (7.9)	62 (8.9)	122 (8.5)
3	47 (6.3)	32 (4.6)	79 (5.5)
>3	55 (7.4)	43 (6.2)	98 (6.8)
Total	743 (100.0)	695 (100.0)	1440 (100.0)
GINA classification			
Step 1	316 (42.5)	0 (0.0)	316 (21.9)
Step 2	427 (57.5)	0 (0.0)	427 (29.7)
Step 3	0 (0.0)	371 (53.4)	371 (25.8)
Step 4	0 (0.0)	261 (37.6)	261 (18.1)
Step 5	0 (0.0)	63 (9.1)	63 (4.4)
Missing data	0 (0.0)	0 (0.0)	2 (0.1)
Total	743 (100.0)	695 (100.0)	1440 (100.0)
Level of asthma control			
Well controlled	318 (42.8)	282 (40.6)	601 (41.7)
Partly controlled	244 (32.8)	258 (37.1)	503 (34.9)
Uncontrolled	181 (24.4)	155 (22.3)	336 (23.3)
Total	743 (100.0)	695 (100.0)	1440 (100.0)
Number of comorbidities			
0	328 (44.1)	264 (38)	593 (41.2)
1–2	319 (42.9)	276 (39.7)	596 (41.4)
3–4	89 (12)	126 (18.1)	215 (14.9)
≥5	7 (0.9)	29 (4.2)	36 (2.5)

Data are presented as *n* (%) unless otherwise specified. ^a^Investigator-classified asthma severity was guided by GINA 2017 treatment steps. Investigators were guided by GINA 2017 treatment steps, either in the study protocol or via a pop-up window in the eCRF.

*eCRF* electronic case report form, *GINA* Global Initiative for Asthma, *max* maximum, *min* minimum, *PCP* primary care physician, *SABINA* SABA use IN Asthma, *SD* standard deviation.

A similar proportion of patients with mild and moderate-to-severe asthma experienced ≥1 (39.0 and 38.4%, respectively) and ≥3 (13.7 and 10.8%, respectively) severe asthma exacerbations in the previous 12 months. The level of asthma symptom control was assessed as well controlled in 41.7%, partly controlled in 34.9%, and uncontrolled in 23.3% of patients.

### SABA prescriptions

**Fig. 1:**
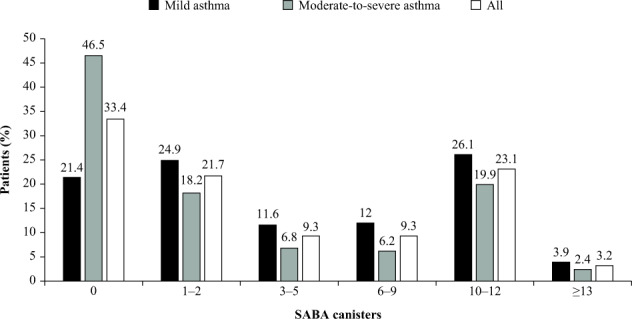
Proportion of patients (%) in the SABINA III PCP cohort (*N* = 1440) receiving SABA prescriptions in the 12 months before the study visit according to investigator-classified asthma severity^a^ The category of patients classified as having 0 SABA canister prescriptions included patients using non-SABA relievers, non-inhaler forms of SABA, and/or SABA purchased OTC. ^a^Investigator-classified asthma severity was guided by GINA 2017 treatment steps. Investigators were guided by GINA 2017 treatment steps either in the study protocol or via a pop-up window in the eCRF. Missing data: *n* = 4, mild asthma; *n* = 31, moderate-to-severe asthma. *eCRF* electronic case report form, *GINA* Global Initiative for Asthma, *OTC* over the counter, *PCP* primary care physician, *SABA* short-acting β_2_-agonist, *SABINA* SABA use IN Asthma.

Overall, 44.9% of patients received prescriptions for ≥3 SABA canisters in the 12 months prior to study entry, and 26.3% received prescriptions for ≥10 SABA canisters; 33.4% of patients were not prescribed any SABA canisters (Fig. 1). Prescription of ≥3 and ≥10 SABA canisters was higher in patients with mild asthma (53.7 and 30.0%, respectively) than in those with moderate-to-severe asthma (35.2 and 22.3%, respectively).

**Fig. 2:**
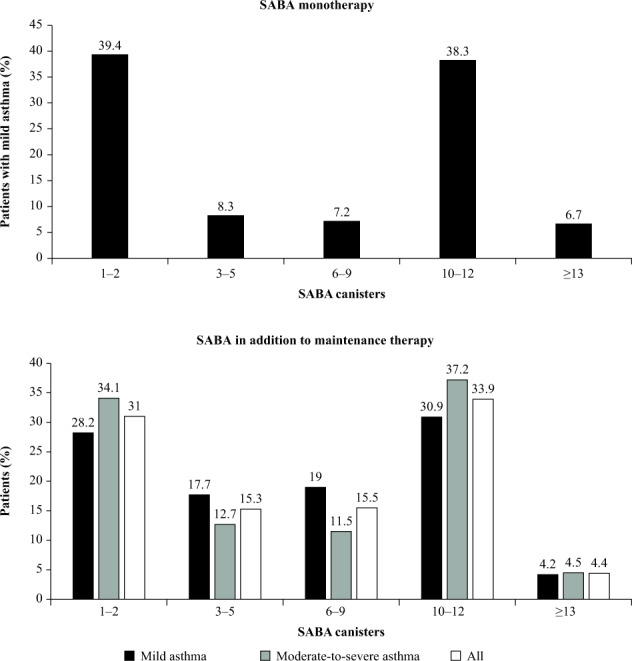
Proportion of patients (%) in the SABINA III PCP cohort (*N* = 1440) receiving prescriptions for SABA monotherapy^a^ and SABA in addition to maintenance therapy in the 12 months prior to study entry according to investigator-classified asthma severity^b^. ^a^SABA monotherapy was prescribed only to patients with mild asthma. ^b^Investigator-classified asthma severity was guided by GINA 2017 treatment steps. Investigators were guided by GINA 2017 treatment steps either in the study protocol or via a pop-up window in the eCRF. Missing data: *n* = 3, patients with mild asthma prescribed SABA alone; *n* = 1, patient with mild asthma prescribed SABA in addition to maintenance treatment; *n* = 31, patients with moderate-to-severe asthma prescribed SABA in addition to maintenance treatment. *eCRF* electronic case report form, *GINA* Global Initiative for Asthma, *PCP* primary care physician, *SABA* short-acting β_2_-agonist, *SABINA* SABA use IN Asthma.

Altogether, 12.7% of patients were prescribed SABA monotherapy, all of whom had mild asthma (Table 3). Among these patients, 60.6 and 45.0% were prescribed ≥3 and ≥10 SABA canisters in the 12 months prior to the study visit, respectively (Fig. 2). More than half of all patients (54.7%) were prescribed SABA in addition to maintenance therapy in the previous 12 months (Table 3). Among these patients, 69 and 38.2% were prescribed ≥3 and ≥10 SABA canisters, respectively (Fig. 2). A comparable proportion of patients with mild and moderate-to-severe asthma were prescribed SABA in addition to maintenance therapy (55.5 and 54.7%, respectively). Prescription of ≥3 SABA canisters was also comparable among patients with mild and moderate-severe asthma (71.8 and 65.9%, respectively).

**Table 3:** Patients in the SABINA III PCP cohort who received prescriptions for (A) SABA monotherapy and (B) SABA in addition to maintenance therapy in the 12 months before the study visit.

	Primary care (*n* = 1440)		
	Investigator-classified mild asthma^a^ (*n* = 743)	Investigator-classified moderate-to-severe asthma^a^ (*n* = 695)	All (*n* = 1440)
A. Patients prescribed SABA monotherapy			
Yes	183 (24.6)	0 (0.0)	183 (12.7)
No	560 (75.4)	695 (100.0)	1257 (87.3)
Number of canisters or inhalers per patient prescribed 12 months before the study visit			
Number of patients	180	NA	180
Mean (SD)	7.2 (6.1)	NA	7.2 (6.1)
Median (min–max)	6.0 (1.0–42.0)	NA	6.0 (1.0–42.0)
Missing values	3 (1.7)	NA	3 (1.7)
B. Patients prescribed SABA in addition to maintenance therapy			
Yes	402 (54.1)	386 (55.5)	788 (54.7)
No	341 (45.9)	309 (44.5)	652 (45.3)
Number of canisters or inhalers per patient prescribed 12 months before the study visit			
Number of patients	401	355	756
Mean (SD)	6.7 (4.7)	8.1 (14.1)	7.4 (10.3)
Median (min–max)	6.0 (1.0–24.0)	6.0 (1.0–210.0)	6.0 (1.0–210.0)
Missing values	1 (0.2)	31 (8.0)	32 (4.1)

Data are presented as *n* (%) unless otherwise specified. ^a^Investigator-classified asthma severity was guided by GINA 2017 treatment steps. Investigators were guided by GINA 2017 treatment steps, either in the study protocol or via a pop-up window in the eCRF.

*eCRF* electronic case report form, *GINA* Global Initiative for Asthm, *max* maximum, *min* minimum, *NA* not available, *PCP* primary care physician, *SABA* short-acting β_2_-agonist, *SABINA* SABA use IN Asthma, *SD* standard deviation.

### SABA obtained OTC without prescriptions

In those countries where SABA was available OTC without a prescription, 21.5% of patients purchased SABA OTC, of whom 40.5 and 6.5% purchased ≥3 and ≥10 SABA canisters, respectively. Among patients who purchased SABA OTC (*n* = 309), 57.9% had received SABA prescriptions as well (Supplementary Fig. 1); 72.7% for ≥3 canisters and 39.1% for ≥10 canisters in the previous 12 months.

### Prescriptions for asthma medications other than SABA

Overall, 31% of patients were prescribed maintenance therapy in the form of ICS, with a mean (SD) of 7.2 (7.0) ICS canisters in the preceding 12 months (Table 4A). An ICS/long-acting β_2_-agonist (LABA) was prescribed to 49.4% of patients (Table 4B).

**Table 4:** Patients in the SABINA III PCP cohort who received prescriptions for (A) ICS, (B) ICS/LABA (fixed-dose combination), and (C) OCS burst/short course in the 12 months prior to study entry.

	Primary care (*n* = 1440)		
	Investigator-classified mild asthma^a^ (*n* = 743)	Investigator-classified moderate-to-severe asthma^a^ (*n* = 695)	All (*n* = 1440)
A. Patients prescribed ICS			
Yes	403 (54.2)	44 (6.3)	447 (31.0)
No	340 (45.8)	651 (93.7)	993 (69.0)
Total prescribed daily ICS dose			
Low dose	132 (32.9)	10 (25.6)	142 (32.3)
Medium dose	231 (57.6)	27 (69.2)	258 (58.6)
High dose	38 (9.5)	2 (5.1)	40 (9.1)
Missing values	2	5	7
Total	401	39	440
Number of canisters or inhalers per patient prescribed 12 months before the study visit			
Number of patients	402	39	441
Mean (SD)	7.5 (7.2)	4.8 (4.1)	7.2 (7.0)
Median (min–max)	6.0 (1.0–110.0)	4.0 (1.0–17.0)	6.0 (1.0–110.0)
Missing values	1 (0.2)	5 (12.8)	6 (1.4)
B. Patients prescribed ICS/LABA (fixed-dose combination)			
Yes	34 (4.6)	675 (97.3)	711 (49.4)
No	709 (95.4)	19 (2.7)	728 (50.6)
Total prescribed daily ICS dose			
Low dose	26 (78.8)	324 (48.2)	350 (49.5)
Medium dose	5 (15.2)	279 (41.5)	286 (40.5)
High dose	2 (6.1)	69 (10.3)	71 (10.0)
Missing values	1	3	4
Total	33	672	707
C. Patients prescribed OCS burst/short course			
Yes	152 (20.5)	157 (22.6)	309 (21.5)
No	590 (79.5)	537 (77.4)	1129 (78.5)
Missing values	1	1	2
Total	742	694	1438

Data are presented as *n* (%) unless otherwise specified. ^a^Investigator-classified asthma severity was guided by GINA 2017 treatment steps. Investigators were guided by GINA 2017 treatment steps, either in the study protocol or via a pop-up window in the eCRF.

*eCRF* electronic case report form, *GINA* Global Initiative for Asthma, *ICS* inhaled corticosteroids, *LABA* long-acting β_2_-agonist, *max* maximum, *min* minimum, *OCS* oral corticosteroids, *PCP* primary care physician, *SABINA* SABA use IN Asthma, *SD* standard deviation.

In the 12 months prior to study entry, 21.5% of patients were prescribed OCS burst treatment (Table 4C). OCS burst prescriptions were common across all SABA categories and asthma severities, regardless of asthma treatments prescribed in the previous 12 months (Supplementary Fig 2). Overall, across most SABA prescription categories (1–2, 3–5, 6–9, and 10–12 canisters), a higher proportion of patients with mild asthma (ranging from 12 to 17.7%) were prescribed OCS bursts compared with those with moderate-to-severe asthma (ranging from 4.3 to 15.1%).

### Association of SABA prescriptions with asthma-related health outcomes

**Fig. 3:**
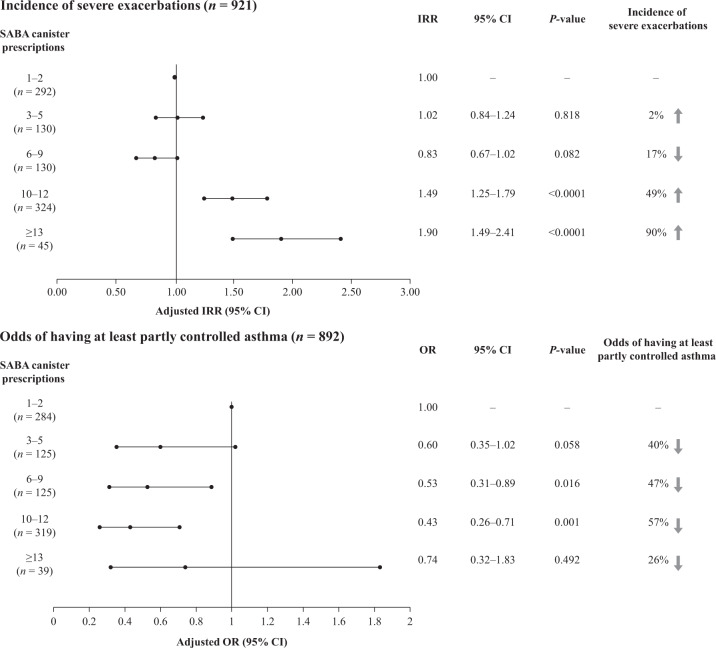
Association of SABA prescriptions with incidence of severe exacerbations in the 12 months prior to study entry and level of asthma control assessed during the study visit in the SABINA III PCP cohort. Note: ORs were adjusted for age, country, sex, and smoking as pre-specified covariates; and GINA step by investigator, healthcare insurance, education level, comorbidities, duration of asthma, and BMI as potential confounders. IRRs were adjusted for age, country, sex, and smoking as pre-specified covariates; and duration of asthma and BMI as potential confounders. *BMI* body mass index, *CI* confidence interval, *GINA* Global Initiative for Asthma, *IRR* incidence rate ratio, *OR* odds ratio, *PCP* primary care physician, *SABA* short-acting β_2_-agonist, *SABINA* SABA use IN Asthma.

Disposition of patients included in the secondary analysis is shown in Supplementary Fig 3. The pre-specified regression analyses, adjusted for pre-specified covariates and potential confounders, showed that prescription of 10–12 and ≥13 SABA canisters (vs 1–2 canisters) in the previous 12 months was associated with a 49% (adjusted incidence rate ratio [IRR], 1.49; 95% confidence interval [CI], 1.25–1.79; *P* < 0.0001) and 90% (adjusted IRR, 1.90; 95% CI, 1.49–2.41; *P* < 0.0001) significantly increased incidence rate of severe exacerbations, respectively. Further details are provided in Fig. 3.

Compared with prescription of 1–2 SABA canisters, prescription of 6–9 and 10–12 SABA canisters in the previous 12 months was associated with a 47% (odds ratio [OR], 0.53; 95% CI, 0.31–0.89; *P* = 0.016) and 57% (OR, 0.43; 95% CI, 0.26–0.71; *P* = 0.001) significantly lower odds of having at least partly controlled asthma (partly controlled plus well-controlled asthma), respectively. Further details are provided in Fig. 3.

### Key differences between the SABINA III PCP cohort and the overall SABINA III population

Data were generally consistent between the two populations, although there were a few exceptions (Supplementary Table 1). Compared with the overall SABINA III population, the PCP cohort had more patients with no healthcare reimbursement (35.5% vs 27.3%). Moreover, fewer patients treated by PCPs, compared with the overall SABINA III population, had ≥1 severe asthma exacerbation (38.8% vs 45.4%). In contrast to the high proportion of patients with moderate-to-severe asthma in the overall SABINA III population (76.5%)^27^, the PCP cohort had a more balanced distribution (mild asthma, 51.7%; moderate-to-severe asthma, 48.3%).

Of those patients prescribed SABA monotherapy, a higher proportion in the PCP cohort compared with the overall SABINA III population^27^ were prescribed ≥3 (60.6% vs 53.6%) and ≥10 (45.0% vs 29.9%) SABA canisters in the 12 months prior to study entry. Although prescriptions for SABA in addition to maintenance therapy were comparable between the PCP cohort and overall SABINA III population (54.7% vs 58.0%, respectively)^27^, a higher proportion of patients treated in primary care were prescribed ≥3 (69% vs 61.7%) and ≥10 (38.2% vs 29.3%) SABA canisters.

## Discussion

This large cross-sectional study in 24 countries provides valuable real-world insights into the management of asthma in primary care at a global scale. Although most patients were prescribed maintenance therapy in the form of ICS or ICS/LABA fixed-dose combination, SABA over-prescription was common, even in patients with mild asthma. Approximately half (44.9%) of all patients were prescribed SABA in excess of current treatment recommendations (≥3 SABA canisters in the previous 12 months), which was associated with poor asthma-related health outcomes, both in terms of increased incidence of severe exacerbations and poor asthma control.

In line with previous studies^19,20^, SABA over-prescription in primary care was high, with a greater proportion of patients in the PCP cohort being prescribed ≥3 and ≥10 SABA canisters (both as monotherapy and in addition to maintenance therapy) compared with the overall SABINA III population^27^. Notably, SABA over-prescription was more common in patients with mild asthma, suggesting potential under-estimation of asthma severity in patients with milder disease or the inappropriate management of patients with ‘mild’ asthma, resulting in poor symptom control.

Additionally, OTC purchase of SABA was common in primary care, with 21.5% of patients purchasing SABA OTC without a prescription in the preceding 12 months. Among patients with both SABA OTC purchase and prescriptions, most had already been prescribed ≥3 (72.7%) and ≥10 (39.1%) canisters, a trend that was more apparent in patients with mild than moderate-to-severe asthma (24.1% vs 18.4%). Since SABA OTC purchase has been associated with under-treatment of asthma^28^, healthcare policy makers and national governments should collaborate effectively to improve access to affordable care and regulate SABA OTC purchase to ensure optimal asthma care.

The prescription of OCS bursts was common across all SABA prescription categories and asthma severities, especially in patients prescribed 1–2 SABA canisters and those with mild asthma. SABA overuse and high exposure to OCS have been associated with insufficient prescription of maintenance therapy^29^, whilst increasing OCS prescriptions (≥4 per year) is associated with deleterious adverse events regardless of dose, duration, or continuous sporadic use^30^. Hence, there is an urgent need to reduce inappropriate prescribing of both SABA and OCS and to accurately document prescriptions, dispensing and use of OCS for worsening asthma symptoms, and managing exacerbations. To this end, in those countries with adequate healthcare services and where physicians have sufficient time to engage in behaviour change counselling, PCPs could provide patients with self-management training to help them recognise symptom worsening and provide instructions on appropriate use of reliever and maintenance medication, as well as OCS^3^.

Findings from this PCP cohort revealed that higher SABA prescriptions were, with a few exceptions, significantly associated with an increase in the rate of severe exacerbations (10–12 and ≥13 canisters) and lower odds of achieving at least partly controlled asthma (6–9 and 10–12 canisters). Although results from the overall SABINA III study showed a statistically significant association between increasing SABA prescriptions and poor asthma-related outcomes across all SABA categories^27^, this was not observed in this PCP cohort, likely due to the smaller patient population. However, findings from a larger cohort of patients (>570,000) in the United Kingdom (UK) who were treated in primary care (SABINA I)^18^ were consistent with those reported in SABINA III. Nonetheless, SABA prescription patterns in the PCP cohort were in line with the overall SABINA III population, highlighting the importance of monitoring both SABA prescriptions and SABA use to identify patients at increased risk of exacerbations, especially those under-prescribed ICS. Indeed, over-prescription of SABAs and insufficient provision of ICS-containing treatments have been identified as preventable causes of death from asthma^16^. Following the National Review of Asthma Deaths (NRAD) in the UK, the Royal College of Physicians (London) in its report titled ‘Why asthma still kills’ recommends that PCPs have an oversight of the patient’s entire prescription history so that SABA over-prescription can be closely monitored^31^.

Most patients treated in primary care were prescribed maintenance medication, either ICS or fixed-dose combinations of ICS/LABA. However, patients were prescribed a mean of 7.2 canisters of ICS in the previous 12 months. This quantity suggests potential under-prescription on the basis that one canister per month is considered good clinical practice^3^, and most patients were not prescribed multiple maintenance treatments, although in some cases, single ICS inhalers provide a ≥2-month supply. In addition, the majority of patients with mild asthma were prescribed ICS at a medium dose (57.6%) instead of the recommended low dose^3^. Taken together, these findings demonstrate the need for better alignment of both reliever and maintenance medication prescription practices with GINA recommendations by adapting asthma management guidelines to the primary care setting. Most currently available guidelines are complex, long, and generally biased towards a secondary care perspective^7^, which may limit their utility and has led the GINA committee to acknowledge the difficulty in implementing their recommendations in primary care^3^; therefore, their adaptation for PCPs to ensure wider acceptance^22,32^ would be of considerable value.

Consistent with earlier studies^33–36^, the level of asthma control in patients treated by PCPs was poor, suggesting that identification of patients with suboptimal asthma control remains a challenge in primary care. Our findings revealed that 57.2% of patients with mild disease reported having uncontrolled/partly controlled asthma, potentially due to inadequate treatment, and suggestive of under-recognition of both disease control and underlying asthma severity. Indeed, both PCPs^33,37,38^ and patients^5^ tend to overestimate the level of asthma control, leading to under-treatment of the disease^37^. Moreover, patients often perceive control as symptom relief and/or management of exacerbations, reflective of crisis-oriented disease management^39^, which may further contribute to SABA over-reliance. This could also account for the high SABA OTC purchase observed in this cohort of patients treated by PCPs, particularly in those with mild asthma. Consequently, use of objective and validated tools, such as the Asthma Control Test (ACT) and the Asthma Control Questionnaire (ACQ)^3^, can assist PCPs in promptly identifying patients who may require a more detailed assessment, thereby addressing the discrepancy between perceived and actual disease control.

Poor asthma control translated into a high disease burden in this PCP cohort, with more than a third of patients (38.8%) experiencing ≥1 severe asthma exacerbation in the preceding 12 months. Moreover, 39.0 and 13.7% of patients with mild asthma experienced ≥1 and ≥3 severe asthma exacerbations, respectively. SABA over-prescription is a modifiable risk factor for exacerbations^40^, with results from a SABINA study conducted in the UK reporting that SABA over-prescription was associated with a 20% increased rate of exacerbations in patients with mild asthma, even after adjusting for various confounding factors^18^. Another possible explanation for this high disease burden is that healthcare reimbursement was not available to a substantial proportion of patients overall (35.5%), including a higher proportion of patients with mild than with moderate-to-severe asthma (43.1% vs 27.5%). Inadequate healthcare insurance coverage and limited access to healthcare continue to be major barriers in accessing cost-effective treatments in many of the countries included in this study^1,41^ and have been associated with a reduced use of regular preventive care, increased use of emergency care^42^, and consistently poorer quality of asthma care, including a lower likelihood of receiving ICS^43^. Furthermore, the high cost of maintenance medication, such as ICS/LABA combination inhalers, can limit its use, especially in low- and middle-income countries where access to affordable medicines represents an unmet need^1,41^.

Overall, our findings reveal that PCPs need to reconsider approaches for managing asthma effectively, particularly in relation to the regular monitoring of both asthma control and SABA prescriptions/use to identify patients at risk of poor health outcomes. Importantly, PCPs need to ensure an accurate evaluation of underlying asthma severity so that appropriate treatment may be initiated or maintained through a step-wise process.

Since many patients with mild asthma remain uncontrolled, frequently have poor treatment adherence and often underestimate the seriousness of their condition, some PCPs could play a role in establishing asthma action plans^25^, discussing treatment-related issues, providing educational support and, if available, referring patients to HCPs trained in asthma education^3^. Although patients recognise early signs of asthma worsening, their initial response is frequently to increase SABA rather than ICS intake. Therefore, symptom-based use of a fast-acting β_2_-agonist and ICS combination as the default reliever option^44^, which relies on patients’ inherent symptom relief–seeking behaviour^45^ and is supported by GINA^3^, offers a viable asthma management strategy to overcome poor adherence since these medications are available in most primary care and low-income settings^44^.

Dispensing/prescription of excess SABA inhalers should be identified as a sign of poorly controlled asthma^31^. Therefore, putting practice systems in place whereby PCPs receive notifications from pharmacies for SABA inhalers supplied without a prescription would serve as a useful reminder to proactively review asthma control^31^. Since PCP consultation times can adversely affect patient care^11^, approaches such as arranging appointments so that they do not occur simultaneously, organising regular reviews in advance, and improving the system for scheduling walk-in patients could further optimise efficiency in the primary care setting^46^.

Since poor asthma-related health outcomes in primary care have been attributed to gaps between evidence-based recommendations and clinical practice^33,47^, further improvements may be achieved by increasing awareness of the latest treatment recommendations and coordinating multidisciplinary care involving a closer collaboration between PCPs and other HCPs^17,48^. While pharmacists can help monitor prescriptions, educate patients on the benefits of ICS-containing medications, and encourage them to seek medical advice before dispensing SABA OTC, address factors such as poor adherence and incorrect inhaler technique, identify patients at risk of suboptimal control and teach self-management strategies to achieve asthma control^17,49^, a greater involvement of specialists can help with referrals^48^, especially following an exacerbation^16^. In addition, quality improvement strategies, including professional development initiatives, audits, and feedback interventions, can assist in closing the gap between best practices and care delivery, thereby improving treatment outcomes^50^ without impacting workload^51^.

Since primary care is the cornerstone of a strong healthcare system^9^, it is essential that PCPs build strong partnerships with patients through shared decision-making, provision of training on self-management^3^ and effective communication skills to increase patient satisfaction, improve health outcomes^3^ and reduce healthcare resource utilisation^52^ without increasing the consultation time^53^. Notably, compared with patients who receive usual care, those who have greater involvement in treatment-related decisions through shared decision-making, where clinicians and patients negotiate a treatment regimen that accommodates patient goals and preferences, report significantly better clinical outcomes, in terms of quality of life, asthma-related healthcare utilisation, rescue medication use, lung function, and the likelihood of well-controlled asthma^54^. Therefore, PCPs can play a major part in encouraging patients to participate in treatment-related decisions and communicate their expectations and concerns^3^.

This study is not without limitations. Prescription data were used as a surrogate for actual medication use and do not provide information on medication adherence, potentially leading to an under-estimation or over-estimation of SABA use. Although OCS bursts were likely prescribed for managing asthma exacerbations, the association between SABA prescriptions and OCS bursts was not analysed. As asthma severity was classified by investigators (guided by GINA 2017 treatment steps), the underlying disease severity may have been misdiagnosed in some patients. However, a global, 3-year, prospective observational study of patients with asthma and/or chronic obstructive pulmonary disease (COPD) from real-world clinical practice demonstrated that investigator-classified disease severity was associated with several clinical and spirometric factors, including exacerbation history and symptom burden, in patients with asthma^55^. Owing to its observational nature, the study may also be prone to bias, e.g. therapies may be differently prescribed depending on disease severity^56^. In addition, as data for OTC purchase of SABA were self-reported, these findings may also be prone to recall bias^56^. Finally, as patients at many sites were predominantly recruited by specialists, results may not reflect primary care practices across all participating countries. However, aggregated data from 1440 patients across 24 countries, with a balanced distribution of those with mild and moderate-to-severe asthma, provide an understanding of global asthma management practices in the primary care setting.

In conclusion, results from 1440 patients from the SABINA III study treated in primary care reported that SABA prescriptions were common, with approximately one in every two patients being prescribed ≥3 SABA canisters in the 12 months prior to the study visit (defined as over-prescription). Unregulated access to SABA was also common, with over one-fifth of patients purchasing SABA OTC, of whom approximately 40% purchased ≥3 SABA canisters in the previous 12 months. Among patients who purchased SABA OTC, most were already receiving SABA prescriptions, of whom over 70.0% received prescriptions for ≥3 SABA canisters. Higher SABA prescriptions (vs 1–2 canisters) were associated with poor asthma-related health outcomes. Prescription of OCS bursts was common across all SABA prescription categories, even in patients with low SABA prescriptions and mild asthma, regardless of asthma medications prescribed in the preceding 12 months. In addition, the level of asthma control was poor, with less than 50% of patients reporting well controlled asthma. Overall, these findings suggest misdiagnosis of disease severity and inappropriate prescribing practices, both of which are an important public health concern, necessitating professional development initiatives at the primary care level to reduce the burden of asthma. Policy changes to regulate SABA prescriptions and OTC purchase, while ensuring access to asthma medications, may help ensure that clinical practices are aligned with the current evidence-based treatment recommendations. Reducing SABA over-reliance at the primary care level and per current evidence-based guidelines may result in improved clinical outcomes.

## Methods

### Study design

SABINA III was a multi-country, observational, cross-sectional study conducted in 24 countries^27^. Patients were recruited from March 2019 to January 2020. Retrospective data from existing medical records and patient data collected during a study visit were entered into an eCRF. Data on exacerbation history and comorbidities and information on prescriptions for asthma medications were entered in the eCRF by physicians based on patient medical records. At the study visit, physicians also enquired as to whether patients had experienced exacerbations that were not captured in their medical records. Findings from the cohort of patients treated by PCPs are presented here.

The primary objective of the study was to describe SABA prescription patterns in the asthma patient population treated by PCPs at an aggregated multi-country level. The secondary objective was to determine the associations between SABA prescriptions and asthma-related health outcomes. In addition, prescriptions of OCS in patients stratified by SABA prescription categories (1–2 vs 3–5, 6–9, 10–12, and ≥13 canisters), investigator-classified asthma severity, and SABA monotherapy and ICS-containing treatments were evaluated post hoc.

The study was conducted in accordance with the study protocol, the Declaration of Helsinki, and local ethics committees. Informed consent was obtained from all patients or their legal guardians per country regulations.

### Study population

Eligible patients included those aged ≥12 years, with a documented diagnosis of asthma, ≥3 consultations with their HCP, and medical records containing data for ≥12 months prior to the study visit. Patients with a diagnosis of other chronic respiratory diseases, such as COPD, or with an acute or chronic condition that, in the opinion of the investigator, would limit their ability to participate in the study were excluded. Potential study sites were selected using purposive sampling with the aim of obtaining a sample representative of asthma treatment patterns within each participating country by a national coordinator, who also facilitated the selection of investigators.

### Study variables

SABA prescriptions in the 12 months before the study visit were recorded and categorised as 0, 1–2, 3–5, 6–9, 10–12, and ≥13 canisters. Prescription of ≥3 SABA canisters in the previous 12 months was defined as over-prescription^57^. For consistency across the whole SABINA programme, one SABA canister was assumed to contain 150 inhalations^57^. ICS canister prescriptions in the 12 months before the study visit were recorded and expressed according to the average daily dose (low, medium, or high).

Secondary variables included investigator-classified asthma severity (guided by GINA 2017 treatment steps [steps 1−2: mild asthma; steps 3−5: moderate-to-severe asthma] either in the study protocol or via a pop-up window in the eCRF)^40^, asthma duration, and prescriptions for asthma medications in the 12 months prior to the study visit (SABA monotherapy, SABA in addition to maintenance therapy, ICS or fixed-dose combination of ICS/LABAs, and OCS burst treatment [defined as a short course of intravenous corticosteroids or OCS administered for 3−10 days or a single dose of an intramuscular corticosteroid to treat an exacerbation]). All patients were also asked about pharmacy purchases of OTC SABA without a prescription, irrespective of whether individual countries permitted SABA purchase OTC; these data were subsequently entered in the eCRF by the investigator. Other variables, including healthcare insurance (not reimbursed, partially reimbursed, or fully reimbursed), education level (primary and secondary school, high school or university and/or post-graduate education), BMI, number of comorbidities, and smoking status, were also recorded.

### Outcomes

Asthma symptom control was evaluated according to the GINA 2017 assessment for asthma control^40^ and categorised as well controlled, partly controlled, and uncontrolled. Severe exacerbations in the preceding 12 months were based on the American Thoracic Society/European Respiratory Society recommendations^58^ and defined as a deterioration in asthma resulting in hospitalisation or emergency room treatment or the need for intravenous corticosteroids or OCS for ≥3 days or a single dose of an intramuscular corticosteroid.

### Statistical analysis

All analyses were conducted at the overall country-aggregated population level. The association of SABA prescriptions (1–2 vs 3–5, 6–9, 10–12, and ≥13 canisters) in the previous 12 months with the incidence rate of severe exacerbations and the odds of achieving at least partly controlled asthma (uncontrolled asthma as the reference) was analysed using negative binomial and logistic regression models, respectively.

The regression models, based on complete-case analyses, were adjusted for pre-specified covariates and potential confounders (based on the literature and modelling data from SABINA I^18^). Both ORs and IRRs were adjusted for the same pre-specified covariates (age, country, sex, and smoking as pre-specified covariates); however, the adjustments differed in the potential confounders used in the two regression models (GINA step by investigator, healthcare insurance, education level, comorbidities, duration of asthma, and BMI were used for ORs, while duration of asthma and BMI were used for IRRs [age, duration of asthma, and BMI as continuous variables; others as categorical or ordinal variables]). Patients with zero SABA prescriptions were excluded from the analyses as it was not possible to determine the alternative reliever medication used. To ensure that the overall SABINA III study was adequately powered, the aim was to enrol up to 500 patients from each participating country, with 20−25 patients recruited from each participating site. All statistical tests were two-sided at a 5% level of significance and were performed using R statistical software (version 3.6.0).

### Reporting summary

Further information on research design is available in the Nature Research Reporting Summary linked to this article.

## Supplementary information

Supplementary material

Reporting Summary

## Data Availability

Data underlying the findings described in this manuscript may be obtained in accordance with AstraZeneca’s data sharing policy described at https://astrazenecagrouptrials.pharmacm.com/ST/Submission/Disclosure.
