# Supplementary material for: Short-acting β2-agonist prescription patterns for asthma management in the SABINA III primary care cohort
Source: NPJ Prim Care Respir Med. 2022 Sep 29;32:37. doi: 10.1038/s41533-022-00295-7 (PMC9522811; doi:10.1038/s41533-022-00295-7)
Supplement: Supplementary file 1 — Supplementary material [file 41533_2022_295_MOESM1_ESM.pdf]

SUPPLEMENTARY SECTION

Supplementary Figure 1. SABA OTC purchases and prescriptions in patients with asthma in the SABINA III PCP cohort (*n* = 309).

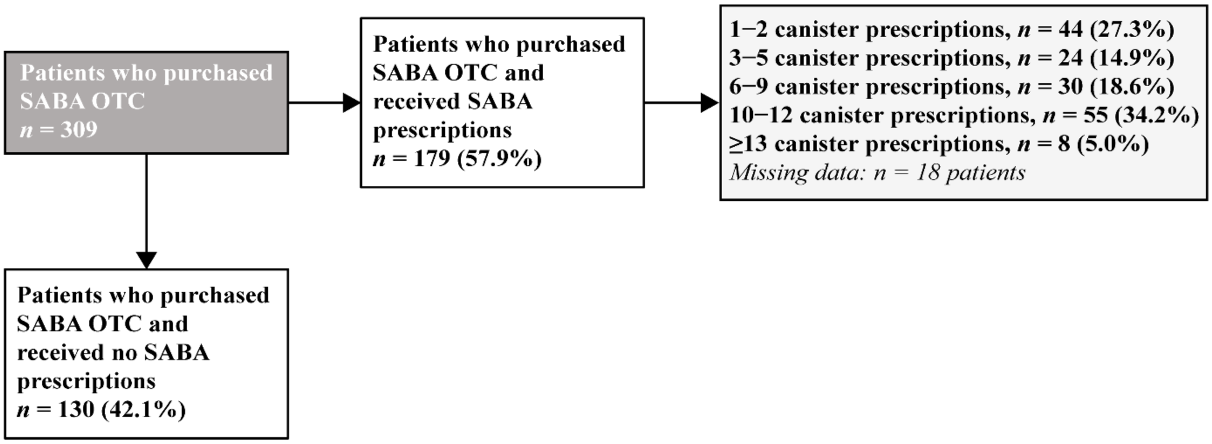

OTC over the counter, *PCP* primary care physician, *SABA* short-acting β_2-agonist, *SABINA* SABA use IN Asthma.

Supplementary Figure 2. OCS burst prescriptions in the SABINA III PCP cohort stratified by SABA prescription categories and investigator-classified asthma severity[a] in overall patients, patients prescribed SABA monotherapy[b], patients prescribed ICS, and patients prescribed ICS/LABA (fixed-dose combination) in the 12 months prior to study entry.

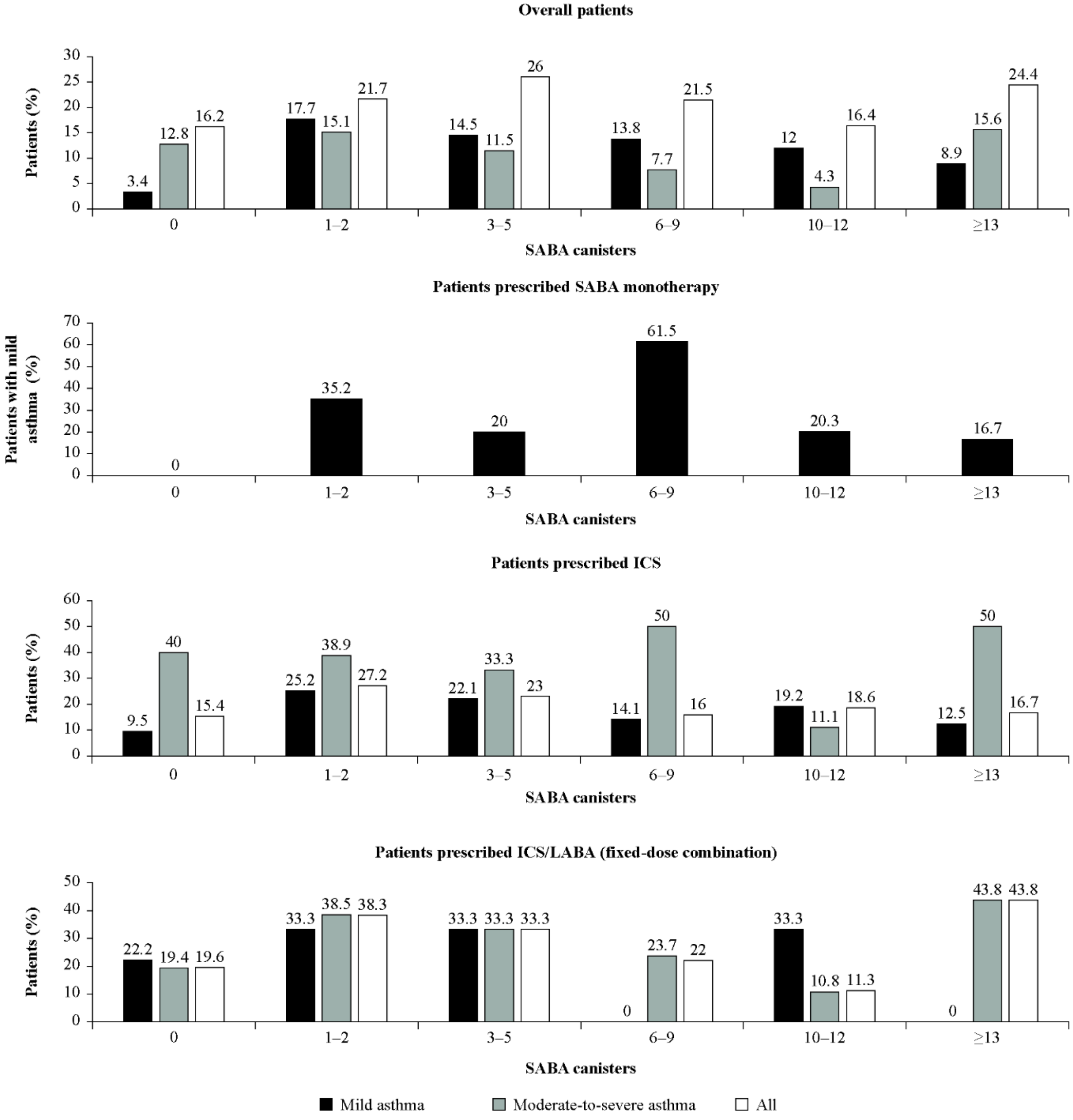

[a]Investigator-classified asthma severity was guided by GINA 2017 treatment steps. [b]OCS bursts were prescribed only to patients with mild asthma receiving SABA monotherapy.

GINA Global Initiative for Asthma, *ICS* inhaled corticosteroids, *LABA* long-acting β_2-agonist, *OCS* oral corticosteroids, *PCP* primary care physician, *SABA* short-acting β_2-agonist, *SABINA* SABA use IN Asthma.

Supplementary Figure 3. Patients from the SABINA III PCP cohort included in the analyses for secondary objectives.

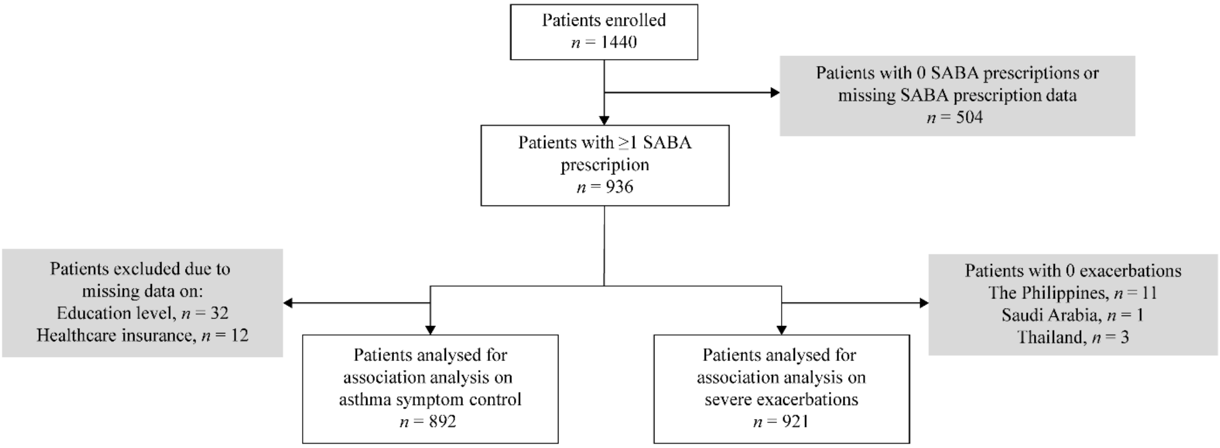

After excluding patients with 0 SABA prescriptions or missing SABA prescription data, all remaining patients from the Philippines (*n* = 11), Saudi Arabia (*n* = 1), and Thailand (*n* = 3) had 0 exacerbations and were excluded from the regression model to analyse the association between SABA and asthma-related outcomes.

PCP primary care physician, *SABA* short-acting β_2-agonist, *SABINA* SABA use IN Asthma.

Supplementary Table 1. Comparison of data between the SABINA III PCP cohort and overall SABINA III population (primary and specialist care).

**Characteristics**		**SABINA III primary care (n = 1,440)**	**Overall SABINA III (n = 8,351)**
**Patient demographics and disease characteristics**			
Age, years	Mean (SD)	47.9 (16.7)	49.4 (16.7)
Sex	Female	988 (68.6)	5691 (68.1)
BMI, kg/m^2	Mean (SD)	27.9 (6.5)	27.8 (6.2)
Smoking status	Active smoker	91 (6.3)	497 (6.0)
	Former smoker	216 (15)	1105 (13.2)
	Never smoker	1133 (78.7)	6747 (80.8)
	Missing data	0 (0.0)	2
Education level	Primary or secondary school	533 (37.0)	2877 (34.5)
	High school	318 (22.1)	2013 (24.1)
	University and/or post-graduate education	501 (34.8)	2792 (33.4)
	Not established	88 (6.1)	668 (8.0)
Healthcare reimbursement	Not reimbursed	511 (35.5)	2281 (27.3)
	Partially reimbursed	348 (24.2)	1851 (22.2)
	Fully reimbursed	539 (37.4)	3940 (47.2)
	Not specified	42 (2.9)	276 (3.3)
	Missing data	0	3
Investigator-classified asthma severity[a]	GINA steps 1–2	743 (51.7)	1958 (23.4)
	GINA steps 3–5	695 (48.3)	6388 (76.5)
	Missing data	2	5
**Asthma-related clinical outcomes 12 months before the study visit**			
Patients with at least 1 severe exacerbation		558 (38.8)	3795 (45.4)
Level of asthma symptom control	Well controlled	601 (41.7)	3610 (43.3)
	Partly controlled	503 (34.9)	2686 (32.2)
	Uncontrolled	336 (23.3)	2034 (24.5)
	Missing data	0	21
**Asthma treatments 12 months before the study visit**			
Patients prescribed SABA monotherapy		183 (12.7)	428 (5.1)
Number of patients prescribed ≥3 SABA canisters		109 (60.6)	226 (53.6)
Number of patients prescribed ≥10 SABA canisters		81 (45.0)	126 (29.9)
Patients prescribed SABA in addition to maintenance therapy		788 (54.7)	4847 (58.0)
Number of patients prescribed ≥3 SABA canisters		522 (69.0)	2869 (61.7)
Number of patients prescribed ≥10 SABA canisters		289 (38.2)	1360 (29.3)
Patients prescribed ICS		447 (31.0)	1473 (17.6)
Patients prescribed ICS/LABA fixed-dose combination		711 (49.4)	6610 (79.2)
Patients prescribed OCS bursts		309 (21.5)	2654 (31.8)

Characteristics	SABINA III primary care (n = 1,440)	Overall SABINA III (n = 8,351)
SABA OTC purchase without prescriptions	309 (21.5)	1503 (18.0)
**Associations between SABA prescriptions and asthma clinical outcomes12 months before the study visit**		
SABA canisters and incidence of severe exacerbations (IRR [95% CI])		
1–2	1.00 (NA)	1.00 (NA)
3–5	1.02 (0.84–1.24)	1.40 (1.24–1.58)
6–9	0.83 (0.67–1.02)	1.52 (1.33–1.74)
10–12	1.49 (1.25–1.79)	1.78 (1.57–2.02)
≥13	1.90 (1.49–2.41)	1.92 (1.61–2.29)
SABA canisters and odds of having at least partly controlled asthma (OR [95% CI])		
1–2	1.00 (NA)	1.00 (NA)
3–5	0.60 (0.35–1.02)	0.64 (0.53–0.78)
6–9	0.53 (0.31–0.89)	0.49 (0.39–0.61)
10–12	0.43 (0.26–0.71)	0.42 (0.34–0.51)
≥13	0.74 (0.32–1.83)	0.33 (0.25–0.45)

Data are presented as *n* (%) unless otherwise specified. [a]Investigator-classified asthma severity was guided by GINA 2017 treatment steps.

BMI body mass index, *CI* confidence interval, *GINA* Global Initiative for Asthma, *ICS* inhaled corticosteroids, *IRR* incidence rate ratio, *LABA* long-acting β_2-agonist, *NA* not available, *OCS* oral corticosteroids, *OTC* over the counter, *OR* odds ratio, *PCP* primary care physician, *SABA* short-acting β_2-agonist, *SABINA* SABA use IN Asthma, *SD* standard deviation.
