# Supplementary material for: Short-acting β2-agonist prescription patterns for asthma management in the SABINA III primary care cohort
Source: NPJ Prim Care Respir Med. 2022 Sep 29;32:37. doi: 10.1038/s41533-022-00295-7 (PMC9522811; doi:10.1038/s41533-022-00295-7)
Supplement: Supplementary file 2 — Reporting Summary [file 41533_2022_295_MOESM2_ESM.pdf]

nature portfolio

Corresponding author(s): David Price

Last updated by author(s): Jun 2, 2022

Reporting Summary

Nature Portfolio wishes to improve the reproducibility of the work that we publish. This form provides structure for consistency and transparency in reporting. For further information on Nature Portfolio policies, see our Editorial Policies and the Editorial Policy Checklist.

Statistics

For all statistical analyses, confirm that the following items are present in the figure legend, table legend, main text, or Methods section.

n/a	Confirmed	
☐	☒	The exact sample size (n) for each experimental group/condition, given as a discrete number and unit of measurement
☒	☐	A statement on whether measurements were taken from distinct samples or whether the same sample was measured repeatedly
☐	☒	The statistical test(s) used AND whether they are one- or two-sided<br>*Only common tests should be described solely by name; describe more complex techniques in the Methods section.*
☐	☒	A description of all covariates tested
☒	☐	A description of any assumptions or corrections, such as tests of normality and adjustment for multiple comparisons
☐	☒	A full description of the statistical parameters including central tendency (e.g. means) or other basic estimates (e.g. regression coefficient) AND variation (e.g. standard deviation) or associated estimates of uncertainty (e.g. confidence intervals)
☒	☐	For null hypothesis testing, the test statistic (e.g. F, t, r) with confidence intervals, effect sizes, degrees of freedom and P value noted<br>*Give P values as exact values whenever suitable.*
☒	☐	For Bayesian analysis, information on the choice of priors and Markov chain Monte Carlo settings
☒	☐	For hierarchical and complex designs, identification of the appropriate level for tests and full reporting of outcomes
☐	☒	Estimates of effect sizes (e.g. Cohen's d, Pearson's r), indicating how they were calculated

Our web collection on statistics for biologists contains articles on many of the points above.

Software and code

Policy information about availability of computer code

Data collection	The methodology for SABINA III, a multi-country, observational, cross-sectional study conducted in 24 countries, has been published previously. Bateman, E. D. et al. Eur Respir J (In Press), doi:0.1183/13993003.01402-2021 (2021). Patients were recruited from March 2019 to January 2020. Retrospective data from existing medical records and patient data collected during a study visit were entered into an eCRF. Findings from the cohort of patients treated by PCPs are presented here.<br>This is currently described in the Methods section.
Data analysis	All analyses were conducted at the overall country-aggregated population level. The association of SABA prescriptions (1–2 vs 3–5, 6–9, 10–12 and ≥13 canisters) in the previous 12 months with the incidence rate of severe exacerbations and the odds of achieving at least partly controlled asthma (uncontrolled asthma as the reference) was analysed using negative binomial and logistic regression models, respectively. The regression models, based on complete-case analyses, were adjusted for pre-specified covariates and potential confounders (based on the literature and modelling data from SABINA I). Both ORs and IRRs were adjusted for the same pre-specified covariates (age, country, sex, and smoking as pre-specified covariates); however, the adjustments differed in the potential confounders used in the two regression models (GINA step by investigator, healthcare insurance, education level, comorbidities, duration of asthma and BMI were used for ORs, while duration of asthma and BMI were used for IRRs [age, duration of asthma and BMI as continuous variable, others as categorical or ordinal variables]). Patients with zero SABA prescriptions were excluded from the analyses as it was not possible to determine the alternative reliever medication used. All statistical tests were two-sided at a 5% level of significance and were performed using R statistical software (version 3.6.0).<br>This is currently described in the Methods section.

For manuscripts utilizing custom algorithms or software that are central to the research but not yet described in published literature, software must be made available to editors and reviewers. We strongly encourage code deposition in a community repository (e.g. GitHub). See the Nature Portfolio guidelines for submitting code & software for further information.

Data

Policy information about availability of data

All manuscripts must include a data availability statement. This statement should provide the following information, where applicable:

- Accession codes, unique identifiers, or web links for publicly available datasets
- A description of any restrictions on data availability
- For clinical datasets or third party data, please ensure that the statement adheres to our policy

Data underlying the findings described in this manuscript may be obtained in accordance with AstraZeneca's data sharing policy described at https://astrazenecagrouptrials.pharmacm.com/ST/Submission/Disclosure.

Field-specific reporting

Please select the one below that is the best fit for your research. If you are not sure, read the appropriate sections before making your selection.

☒ Life sciences ☐ Behavioural & social sciences ☐ Ecological, evolutionary & environmental sciences

For a reference copy of the document with all sections, see nature.com/documents/nr-reporting-summary-flat.pdf

Life sciences study design

All studies must disclose on these points even when the disclosure is negative.

Sample size	To ensure that the overall SABINA III study was adequately powered, the aim was to enroll up to 500 patients from each participating country, with 20–25 patients recruited from each participating site.
Data exclusions	For the analysis of association between SABA prescriptions and asthma-related outcomes, patients with zero SABA prescriptions were excluded as it was not possible to determine the alternative reliever medication used. This further explained in supplementary figure 3.
Replication	Not applicable. This was a real-world observational study.
Randomization	Not applicable. This was a real-world observational study.
Blinding	Not applicable. This was a real-world observational study.

Reporting for specific materials, systems and methods

We require information from authors about some types of materials, experimental systems and methods used in many studies. Here, indicate whether each material, system or method listed is relevant to your study. If you are not sure if a list item applies to your research, read the appropriate section before selecting a response.

Materials & experimental systems

n/a	Involved in the study	
☒	☐	Antibodies
☒	☐	Eukaryotic cell lines
☒	☐	Palaeontology and archaeology
☒	☐	Animals and other organisms
☐	☒	Human research participants
☒	☐	Clinical data
☒	☐	Dual use research of concern

Methods

n/a	Involved in the study	
☒	☐	ChIP-seq
☒	☐	Flow cytometry
☒	☐	MRI-based neuroimaging

Human research participants

Policy information about studies involving human research participants

Population characteristics	The details are currently described in the Results section.
Recruitment	The methodology for SABINA III, a multi-country, observational, cross-sectional study conducted in 24 countries, has been published previously (Bateman, E. D. et al. Eur Respir J (In Press), doi:0.1183/13993003.01402-2021 (2021). Patients were recruited from March 2019 to January 2020. Retrospective data from existing medical records and patient data collected during a study visit were entered into an eCRF. Findings from the cohort of patients treated by PCPs are presented here.

Ethics oversight	The study was conducted in accordance with the study protocol, the Declaration of Helsinki and local ethics committees.

Note that full information on the approval of the study protocol must also be provided in the manuscript.
